# Using AI to Translate and Simplify Spanish Orthopedic Medical Text: Instrument Validation Study

**DOI:** 10.2196/70222

**Published:** 2025-03-21

**Authors:** Saman Andalib, Aidin Spina, Bryce Picton, Sean S Solomon, John A Scolaro, Ariana M Nelson

**Affiliations:** 1 UCI School of Medicine, University of California, 1001 Health Sciences Rd, Irvine, CA, 92617, United States, 1 (949) 824-6119; 2 Department of Orthopaedic Surgery, UC Irvine Health, Orange, United States; 3 Department of Anesthesiology, UC Irvine Health, Orange, United States

**Keywords:** large language models, LLM, patient education, translation, bilingual evaluation understudy, GPT-4, Google Translate

## Abstract

**Background:**

Language barriers contribute significantly to health care disparities in the United States, where a sizable proportion of patients are exclusively Spanish speakers. In orthopedic surgery, such barriers impact both patients’ comprehension of and patients’ engagement with available resources. Studies have explored the utility of large language models (LLMs) for medical translation but have yet to robustly evaluate artificial intelligence (AI)–driven translation and simplification of orthopedic materials for Spanish speakers.

**Objective:**

This study used the bilingual evaluation understudy (BLEU) method to assess translation quality and investigated the ability of AI to simplify patient education materials (PEMs) in Spanish.

**Methods:**

PEMs (n=78) from the American Academy of Orthopaedic Surgery were translated from English to Spanish, using 2 LLMs (GPT-4 and Google Translate). The BLEU methodology was applied to compare AI translations with professionally human-translated PEMs. The Friedman test and Dunn multiple comparisons test were used to statistically quantify differences in translation quality. A readability analysis and feature analysis were subsequently performed to evaluate text simplification success and the impact of English text features on BLEU scores. The capability of an LLM to simplify medical language written in Spanish was also assessed.

**Results:**

As measured by BLEU scores, GPT-4 showed moderate success in translating PEMs into Spanish but was less successful than Google Translate. Simplified PEMs demonstrated improved readability when compared to original versions (*P*<.001) but were unable to reach the targeted grade level for simplification. The feature analysis revealed that the total number of syllables and average number of syllables per sentence had the highest impact on BLEU scores. GPT-4 was able to significantly reduce the complexity of medical text written in Spanish (*P*<.001).

**Conclusions:**

Although Google Translate outperformed GPT-4 in translation accuracy, LLMs, such as GPT-4, may provide significant utility in translating medical texts into Spanish and simplifying such texts. We recommend considering a dual approach—using Google Translate for translation and GPT-4 for simplification—to improve medical information accessibility and orthopedic surgery education among Spanish-speaking patients.

## Introduction

It has been well documented that racial and ethnic minority patient groups in the United States endure substantial limitations in patient care [1]. Specifically, significant disparities in health care outcomes between White populations and Hispanic populations persist in several overarching domains of medicine, including but not limited to rates of diabetes, hypertension, and insurance status [2]. Moreover, previous research suggests that language barriers may be associated with larger lapses in perioperative process-of-care outcomes [3], and patient populations who experience language barriers also face increased predisposition to hospital readmission and emergency department visits, further highlighting their susceptibility to undesired health care outcomes [4].

In the field of orthopedic surgery, these disparities are broadly evident [5-7]. From initial access to orthopedic care to postoperative outcomes, Spanish-speaking patients contend with significant barriers in accessing high-quality care [6,7]. Hispanic populations often have limitations in their ability to schedule appointments for orthopedic concerns and often do not pursue revision surgery in cases of nonoptimal outcomes after surgical intervention [7,8]. During orthopedic clinic visits, more than half of Spanish-speaking patients have been asked to rely on nonqualified or ad hoc interpreters rather than professional services, indicating that this patient group faces limitations in access to clear and accurate information about orthopedic procedures and services [9]. These disparities may interact and thereby have implications on patient-reported outcome measures (PROMs) for Spanish-speaking populations. Additionally, recent work has evaluated the suitableness of PROMs for Spanish-speaking populations [10]. Commonly used PROMs for Spanish-speaking patient groups were shown to be written at a reading level above the recommended complexity for patient populations in the United States. Technological advancements can provide avenues to address these concerns if they are implemented in a manner that is tailored to their intended patient populations [11,12]. Thus, given the widespread documentation of disparities in orthopedic care that Spanish-speaking patients endure, further evaluation of how emerging technologies can address these lapses is extremely important.

Artificial intelligence (AI) has provided unique solutions to problems in health care, including those related to graduate medical education and patients’ comprehension of medical text [13-17]. Recent work has turned to using publicly available large language models (LLMs) to translate patient discharge summaries and frequently asked questions. The utility of these tools in translating medical text has been illustrated in qualitative textual evaluations conducted via human grading [18,19]. However, studies have yet to evaluate AI-enabled textual translation through robust quantitative analysis involving bilingual evaluation understudy (BLEU) analysis [20]. This methodology quantitatively rates machine-translated text against human translation and has been used in clinical studies [21-23]. Additionally, no study has evaluated AI-driven simplification of Spanish medical text, although AI-driven simplification is a functionality that our group previously quantitatively evaluated for English medical text [16,24,25].

The goals of this study were twofold. First, we aimed to conduct a robust quantitative evaluation of machine translations of medical text by using BLEU analysis, and second, we aimed to assess whether AI platforms can be used to simplify orthopedic medical text written in Spanish.

## Methods

### Study Design

A total of 78 patient education materials (PEMs) from the American Academy of Orthopaedic Surgery (AAOS) were translated from English into Spanish, using 4 different GPT-4 input prompts via the application programming interface (prompts 1‐4; Multimedia Appendix 1) [26] and Google Translate via the googletrans package (SuHun Han). Each machine-generated translation was compared to the professionally human-translated reference from the AAOS, using BLEU analysis via the Natural Language Toolkit (NLTK) [27]; BLEU scores range from 0 to 1, with scores of ≥0.5 indicating high similarity to a designated reference text. A Friedman test, followed by a Dunn multiple comparisons test, was performed for each BLEU score to quantify differences in translation quality. Unigram, bigram, trigram, and fourgram precision analyses were conducted to further assess the translation quality. A Friedman test was followed by Dunn multiple comparisons for each precision metric.

To assess the simplification of the PEMs, we compared the readability of translations generated by GPT-4’s prompt 1 and that of the original AAOS Spanish versions before and after simplification. Spanish text was simplified by using a standardized prompt that was validated for medical use cases [16]. Text complexity was analyzed by counting sentences, words, and syllables with custom functions and the NLTK library [27]. Readability was evaluated by using the Fernández-Huerta readability formula (FH = 206.84 – [0.60 × P] − [1.02 × F]; FH: reading ease score; P: average number of syllables per 100 words; F: average number of sentences per 100 words) [28] and the INFLESZ readability formula (INFLESZ = 206.835 – [62.3 × S/P] – [P/F]; S: total number of syllables; P: total number of words; F: total number of sentences) [29]. The Wilcoxon matched-pairs signed rank test was applied to compare the original and simplified versions, and the Spearman correlation coefficient was used to measure the strength of the association between the simplification process and improved readability.

To assess the impact of original English text features on translation quality, a feature analysis was performed. Random forest regression was completed, using 4 input features (number of words, average number of words per sentence, total number of syllables, and average number of syllables per sentence) of the original English PEM, to predict 20 distinct BLEU scores. These scores encompassed 4 BLEU scoring methods for Google Translate and 4 different GPT-4 input prompts. A 5-fold cross-validation was used to minimize overfitting of the data and to ensure robust feature importance calculations. Average importance scores across all folds were calculated to assess the contribution of each feature for translation performance.

### Ethical Considerations

No application was submitted for review board assessment because no human or animal participants participated directly or indirectly in this study. The University of California, Irvine Institutional Review Board does not require assessment of studies that do not directly or indirectly involve human or animal participants. This study consisted solely of a quantitative evaluation of machine translations and was hence exempt from any institutional review.

## Results

### BLEU Analysis

**Figure 1.**
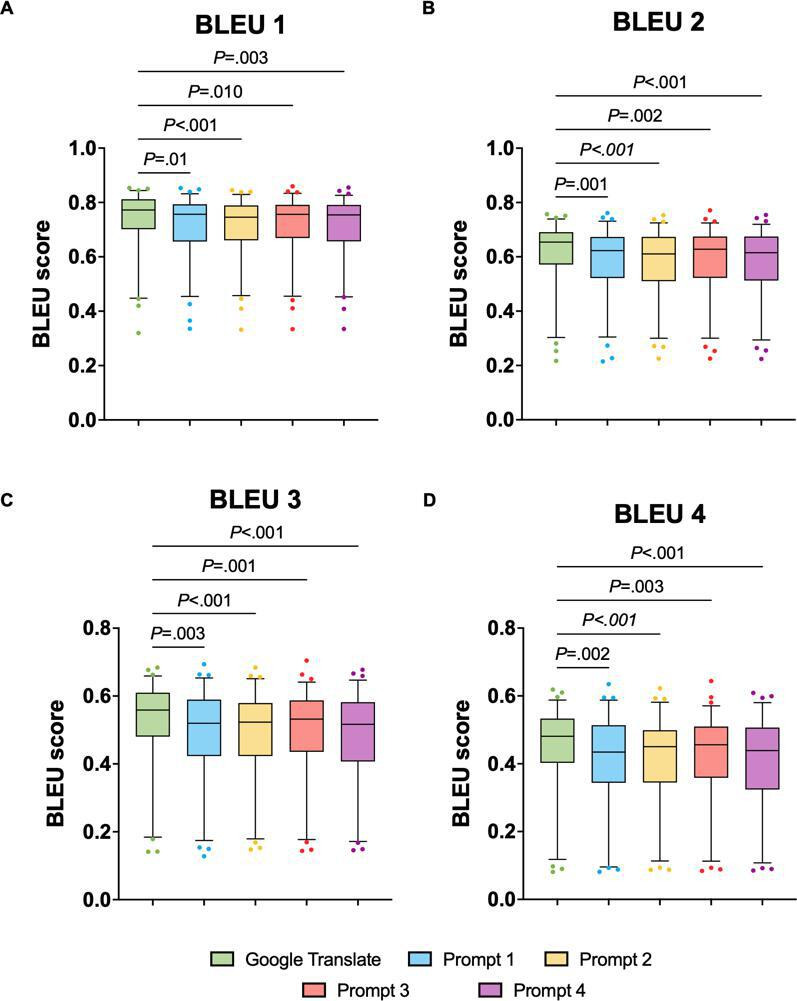
BLEU scores for Google Translate and 4 GPT-4 input prompts (prompts 1-4). Box plots display the BLEU 1 (**A**), BLEU 2 (**B**), BLEU 3 (**C**), and BLEU 4 (**D**) scores for translations generated by Google Translate and the 4 different GPT-4 input prompts. BLEU: bilingual evaluation understudy.

BLEU 1 scores (Figure 1A) revealed a statistically significant difference between Google Translate and each prompt (prompt 1: rank sum difference=63.00; *P*=.01; prompt 2: rank sum difference=81.00; *P*<.001; prompt 3: rank sum difference=65.00; *P*=.01; prompt 4: rank sum difference=71.00; *P*=.003). No significant differences were observed among the 4 GPT prompts (all *P* values were >.05). For BLEU 1, Google Translate had the highest rank sum (290.0), while prompt 2 had the lowest (209.0). Prompt 1 had a rank sum of 227.0, while prompts 3 and 4 had rank sums of 225.0 and 219.0, respectively.

For BLEU 2 scores (Figure 1B), a similar trend was observed, with significant differences between Google Translate and prompts 1, 2, 3, and 4. The rank sum difference was 76.00 between Google Translate and prompt 1 (*P*<.001), 79.00 between prompt 2 and Google Translate (*P*<.001), 73.00 between prompt 3 and Google Translate (*P*=.002), and 77.00 between prompt 4 and Google Translate (*P*<.001). Again, no statistically significant differences were found between the 4 GPT prompts (all *P* values were >.05). The rank sum for Google Translate was the highest (295.0), followed by those for prompt 3 (222.0), prompt 1 (219.0), and prompt 4 (218.0). Prompt 2 had the lowest rank sum (216.0).

For the BLEU 3 scores (Figure 1C), the Dunn test also showed significant differences between Google Translate and each prompt (prompt 1: rank sum difference=72.00; *P*=.003; prompt 2: rank sum difference=85.00; *P*<.001; prompt 3: rank sum difference=76.00; *P*=.001; prompt 4: rank sum difference=82.00; *P*<.001). No significant differences were found between the 4 GPT prompts (all *P* values were >.05). The rank sums were as follows: 297.0 for Google Translate, 225.0 for prompt 1, 212.0 for prompt 2, 221.0 for prompt 3, and 215.0 for prompt 4.

Finally, BLEU 4 scores (Figure 1D) followed the same pattern as the BLEU scores in all 3 prior BLEU analyses, as the Dunn test revealed significant differences between Google Translate and each prompt (prompt 1: rank sum difference=74.00; *P*=.002; prompt 2: rank sum difference=77.00; *P*<.001; prompt 3: rank sum difference=72.00; *P*=.003; prompt 4: rank sum difference=82.00; *P*<.001). Google Translate had the highest rank sum (295.0), followed by prompt 3 (223.0), prompt 1 (221.0), and prompt 2 (218.0). Prompt 4 had the lowest rank sum (213.0).

### N-Gram Precision Analysis

**Figure 2.**
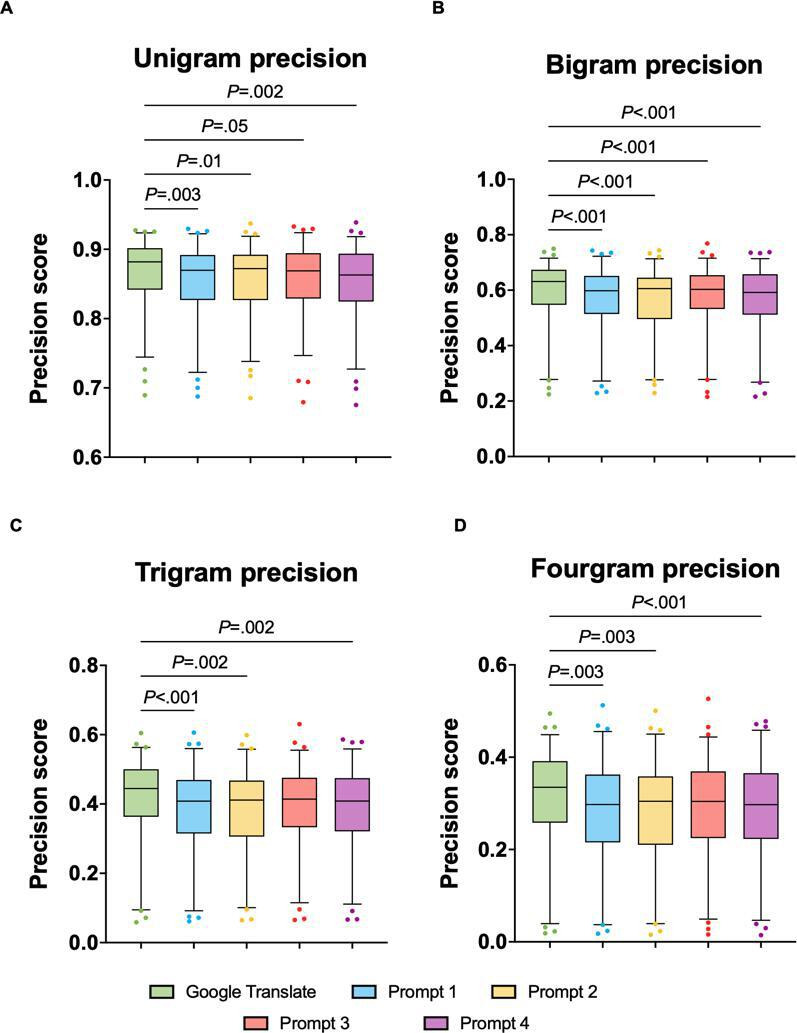
N-gram precision for Google Translate and 4 GPT-4 input prompts (prompts 1-4). Box plots display unigram (**A**), bigram (**B**), trigram (**C**), and fourgram (**D**) precision scores for translations generated by Google Translate and the 4 different GPT-4 input prompts.

The unigram precision analysis (Figure 2A) revealed significant differences between Google Translate and prompts 1, 2, 3, and 4. The rank sum difference was 71.50 between Google Translate and prompt 1 (*P*=.003), 64.00 between prompt 2 and Google Translate (*P*=.01), 55.50 between prompt 3 and Google Translate (*P*=.05), and 74.00 between prompt 4 and Google Translate (*P*=.002). Google Translate had the highest rank sum (287.0), followed by prompt 3 (231.5), prompt 2 (223.0), and prompt 1 (215.5). Prompt 4 had the lowest rank sum (213.0).

The bigram precision analysis (Figure 2B) also revealed significant rank sum differences between Google Translate and each prompt (prompt 1: rank sum difference=93.00; *P*<.001; prompt 2: rank sum difference=88.50; *P*<.001; prompt 3: rank sum difference=79.50; *P*<.001; prompt 4: rank sum difference=99.00; *P*<.001). Google Translate had the highest rank sum (306.0), followed by prompt 3 (226.5). Prompt 2 followed with a rank sum of 217.5, and prompts 1 and 4 had a rank sum of 213.0 and 207.0, respectively.

For the trigram precision analysis (Figure 2C), the Dunn test revealed a pattern that was slightly different from the previously established pattern, with significant differences between Google Translate and prompt 1 (rank sum difference=80.00; *P*<.001), between Google Translate and prompt 2 (rank sum difference=73.00; *P*=.002), and between Google Translate and prompt 4 (rank sum difference=74.00; *P*=.002). There was no significant difference in trigram precision between Google Translate and prompt 3 (*P*=.07). Google Translate had the highest rank sum (290.0), followed by prompt 3 (237.0). Prompt 2 had a rank sum of 217.0, while prompt 4 had a rank sum of 216.0. The lowest rank sum for trigram precision was recorded for prompt 1 (210.0).

The fourgram precision analysis (Figure 2D) showed the same pattern of significance as that in the trigram analysis, with significant differences between Google Translate and GPT prompts 1, 2, and 4. The rank sum difference between Google Translate and prompt 1 was 71.00 (*P*=.003). The rank sum differences between Google Translate and prompt 2 and between Google Translate and prompt 4 were 72.00 (*P*=.003) and 78.00 (*P*<.001), respectively. Fourgram precision showed no statistically significant difference between Google Translate and prompt 3 (*P*=.06). Google Translate had the highest rank sum (289.0), while prompt 3 ranked second with a rank sum of 235.0. Prompt 1 had a rank sum of 218.0, and prompt 2 closely followed with a rank sum of 217.0. Prompt 4 had the lowest rank sum (211.0).

### Simplification Analysis

**Figure 3.**
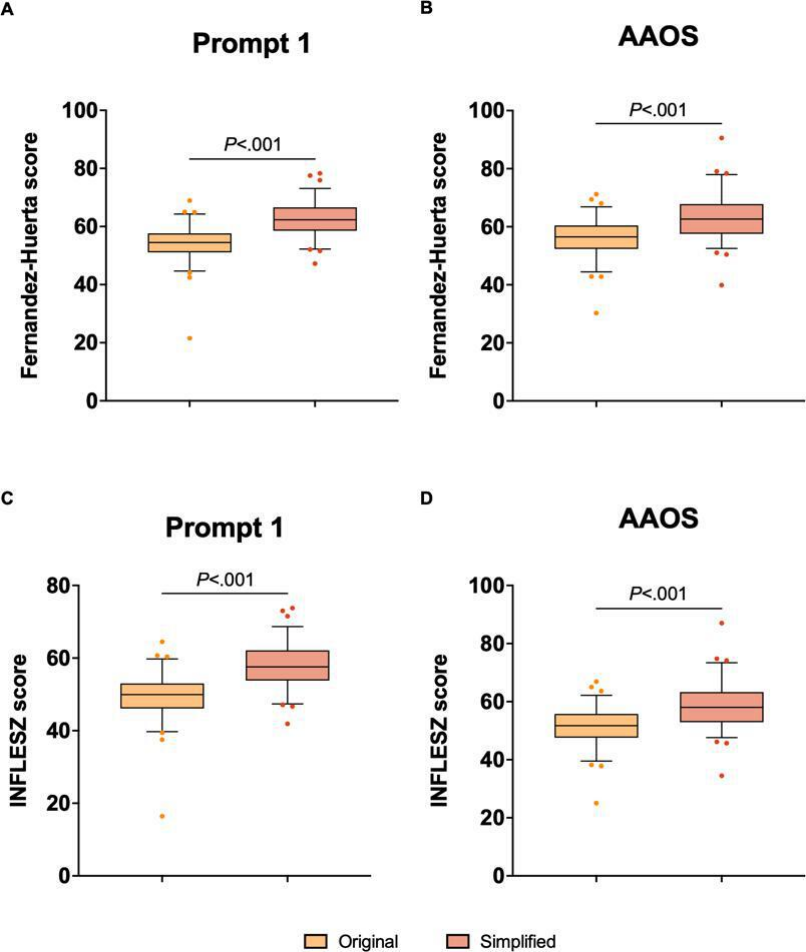
Fernández-Huerta and INFLESZ scores for the original translations by prompt 1 and the AAOS and for their simplified versions. Box plots display the Fernández-Huerta readability scores (**A and B**) and INFLESZ readability scores (**C and D**) for the original and simplified versions of the PEMs generated by GPT-4’s prompt 1 (**A and C**) and for the original and simplified AAOS translations (**B and D**). AAOS: American Academy of Orthopaedic Surgery; PEM: patient education material.

As measured by the Fernández-Huerta scores, the simplified prompt 1 PEM translations and simplified AAOS Spanish PEMs demonstrated significant improvements in readability when compared to the original translations (Figure 3). The Wilcoxon (W) test for prompt 1 showed a significant difference between the original and simplified translations, with a W value of 3059 (*P*<.001); the median difference was 7.846, and the Spearman correlation coefficient was 0.6459 (*P*<.001). For the AAOS Spanish version, the Wilcoxon test revealed a significant improvement after simplification, with a W value of 3055 (*P*<.001) and a median difference of 5.807; the Spearman correlation coefficient was 0.6731 (*P*<.001).

For the INFLESZ scores, similar results were observed. For prompt 1, the Wilcoxon matched-pairs signed rank test indicated a significant difference between the original and simplified translations, with a W value of 3058 (*P*<.001); the median difference was 7.830, and the Spearman correlation coefficient was 0.6591 (*P*<.001). For the AAOS Spanish PEMs, the Wilcoxon test showed a significant improvement after simplification, with a W value of 3045 (*P*<.001) and a median difference of 5.887; the Spearman correlation coefficient was 0.6926 (*P*<.001).

### Feature Analysis

**Figure 4.**
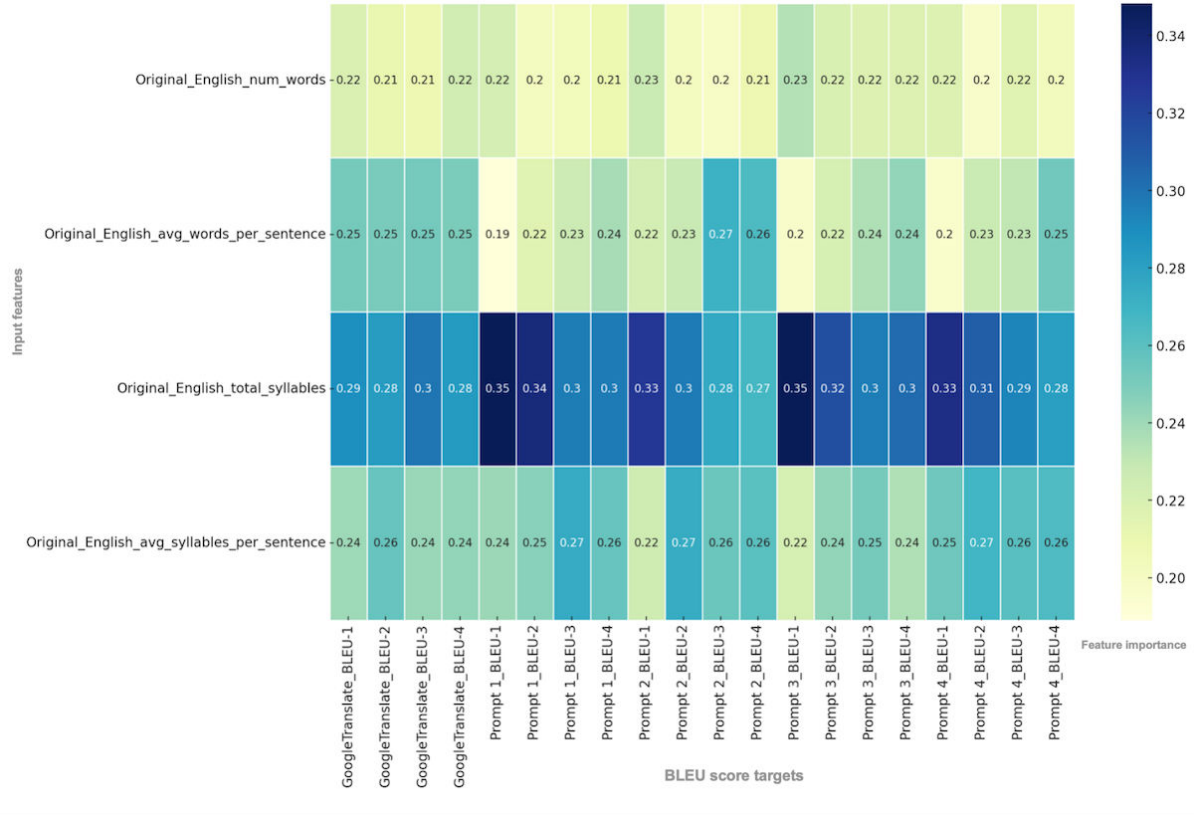
Feature importance scores of English text characteristics for predicting BLEU scores. The heat map shows the relative importance of 4 input features—number of words, average number of words per sentence, total number of syllables, and average number of syllables per sentence—in predicting BLEU scores across the 4 BLEU analyses for each of the 5 translation methods. Darker colors represent higher feature importance. avg: average; BLEU: bilingual evaluation understudy; num: number.

The feature importance analysis of the original English text features revealed that the total number of syllables was the most influential predictor of BLEU scores across Google Translate and GPT-4 prompts, serving as the most important feature (ie, input variable) in every iteration, with scores ranging from 0.27 to 0.35 (Figure 4). The feature importance range for the number of words was 0.2 to 0.23, that for the average number of words per sentence was 0.19 to 0.27, and that for the average number of syllables per sentence was 0.22 to 0.27. Overall, syllable-based features, particularly the total number of syllables, served as the highest-importance features in determining BLEU scores across all translation methods.

## Discussion

### Context

Disparities in communication with Spanish-speaking populations can negatively affect patient education and subsequent outcomes in the field of orthopedic surgery [5-7]. Accurate translation of medical text is one component of properly educating Spanish-speaking patient populations about orthopedic conditions. For orthopedic surgeons, it is vital to ensure that Spanish-speaking patients are properly informed about their conditions and opportunities for surgery, given their increased propensity for hospital readmission, complications, and negative outlooks on surgical intervention [6-8]. Previous work provided a foundation for quantitatively evaluating AI-based medical text translation; however, no study has used BLEU methodology to provide a robust, machine learning–based evaluation of translation success. Additionally, no study has evaluated the AI-enabled simplification of Spanish text. Given the recently outlined need for simplified Spanish text among Spanish-speaking patient populations, this is a pressing need in the field [10]. Our study used a robust corpus of patient-facing orthopedic medical text that included language from across various subspecialties and topics of orthopedic surgery, including the spine, hip, knee, and upper extremities, among others. Through analyzing the success of openly accessible LLMs in translating such text, we aimed to comprehensively assess the translation options available for orthopedic practice.

### Translation Success

This study demonstrated that LLMs, such as ChatGPT, can translate orthopedic PEMs with moderate success, as quantified through BLEU analysis. By experimenting with 4 different model prompts, we explored whether prompt optimization could enhance translation effectiveness. Our findings suggest that while prompt optimization can improve translation outcomes, Google Translate generally provides superior translation quality when compared to human-translated benchmarks. This superior performance highlights the potential of Google Translate for rapid translation tasks, such as translating patient directives in discharge summaries and other patient-facing documents. However, despite its prevalent use, Google Translate’s limitations underscore the need for alternative translation solutions [19,30,31]. The feature analysis conducted within our study also revealed that the syllable complexity of the original English text is a critical predictor of successful translation for both Google Translate and ChatGPT, indicating areas for further refinement in translation approaches. An example AI translation, along with the original English and Spanish versions of the same PEM, can be found in Multimedia Appendix 1.

### Simplification Success

We also assessed the capability of ChatGPT in simplifying medical texts written in Spanish, using a standardized simplification prompting structure that was previously evaluated by our group. Although the platform was able to simplify the text, it did not achieve the targeted grade level specified in our prompts. This limitation aligns with prior studies that highlighted challenges in simplifying English medical texts [16]. However, despite existing challenges with the precision of AI-simplified text in meeting prespecified grade levels, the ability of ChatGPT to simplify texts could greatly benefit Spanish-speaking patients, given that no alternative exists to aid patient comprehension in this way. This is of great importance, considering the complexity of the PROMs and other tools used to assess the operative success of orthopedic procedures in this patient group [10]. Further studies should elucidate ways to best optimize the simplification of Spanish texts via AI platforms.

### Recommendations

Based on our results, we offer several recommendations for orthopedic surgeons. Although Google Translate remains a superior tool for translating English to Spanish due to its adherence to human translation quality, LLMs, such as ChatGPT, also show moderate success and can be considered for specific use cases. Importantly, ChatGPT’s ability to simplify Spanish texts makes it a valuable tool for enhancing patient comprehension and engagement, particularly when translation by a native Spanish speaker is not feasible. We recommend using ChatGPT as an adjunct tool for both translating and simplifying medical texts. Surgeons should continue to use Google Translate for straightforward translations, but they should also consider leveraging ChatGPT’s simplification capabilities to improve the accessibility of medical information. Further research into simplification methodologies is essential for optimizing PROMs and ultimately enhancing patient satisfaction following surgical care. We believe that this technology, once it is fully optimized and vetted, will have the potential to be incorporated into the electronic health record to aid in medical record management through textual translation of records for patients.

### Limitations

This study, while providing insights into the potential of LLMs for translating and simplifying medical texts, has several limitations. First, this study assessed existing models, only tested English-to-Spanish translations, and used a relatively small amount of content, thereby limiting the generalizability of our findings. Second, the BLEU metric, which we used to evaluate translation accuracy, primarily measures literal translation and may not fully capture semantic equivalence, which is critical in medical contexts. Future research could benefit from incorporating additional evaluations that involve human assessment to provide a more nuanced analysis. Third, this study’s focus was on technical performance; we did not directly measure the impact on patient outcomes, such as comprehension, adherence, and satisfaction. Future studies should aim to link the quality of translations and simplifications to specific patient-centered outcomes. Clinical studies would provide valuable insights into the way that Spanish-speaking patient populations interact with and subsequently benefit from AI-enhanced PEMs, such as those analyzed in this study. Lastly, although the corpus of 78 PEMs covered a broad scope of orthopedic literature from all subspecialties, this means that the results of this study only reflect the language used in standard orthopedic practice. Future studies should aim to replicate our results in other medical specialties to provide a broad understanding of the capabilities of AI in translation and simplification.

### Conclusions

This study highlights the utility and limitations of AI-driven tools in translating and simplifying medical texts for Spanish-speaking orthopedic patients. Our findings indicate that while Google Translate provides superior accuracy in translating medical texts, LLMs, such as ChatGPT, demonstrate moderate success and offer significant benefits in simplifying complex medical information into more comprehensible formats. Our recommended dual approach—leveraging Google Translate for accuracy and ChatGPT for simplification—presents a practical solution for enhancing patient education and engagement. Such advancements underscore the potential of AI to bridge the language gap in health care and thereby improve treatment outcomes. Future research should continue to refine these AI tools and enhance their precision and accessibility to meet the diverse needs of patient populations, thereby ensuring that all patients receive care that is both understandable and culturally competent.

## Supplementary material

10.2196/70222 Multimedia Appendix 1 Example artificial intelligence–translated patient education material (PEM) with original English and original Spanish PEMs.

## Abbreviations

AAOS: American Academy of Orthopaedic Surgery
AI: artificial intelligence
BLEU: bilingual evaluation understudy
LLM: large language model
NLTK: Natural Language Toolkit
PEM: patient education material
PROM: patient-reported outcome measure
