# Supplementary material for: Using AI to Translate and Simplify Spanish Orthopedic Medical Text: Instrument Validation Study
Source: JMIR AI. 2025 Mar 21;4:e70222. doi: 10.2196/70222 (PMC12223325; doi:10.2196/70222)
Supplement: Multimedia Appendix 1 [file ai-v4-e70222-s001.docx]

**Original English PEM**

A rotator cuff tear is a common cause of shoulder pain and disability among adults. Each year, almost 2 million people in the United States visit their doctors because of rotator cuff tears.
A torn rotator cuff may weaken your shoulder. This means that many daily activities, like combing your hair or getting dressed, may become painful and difficult to do.
Anatomy

The shoulder is a ball-and-socket joint made up of three bones:
The humerus (upper arm bone)
The scapula (shoulder blade)
The clavicle (collarbone)
The ball, or head, of the upper arm bone fits into a shallow socket in the shoulder blade.
Normal shoulder anatomy
This illustration of the shoulder highlights the major components of the joint.
Your arm is kept in your shoulder socket by the rotator cuff. The rotator cuff is a group of four muscles that come together as tendons to form a covering around the head of the humerus. The rotator cuff attaches the humerus to the shoulder blade and helps to lift and rotate your arm.
The rotator cuff tendons
The rotator cuff tendons cover the head of the humerus (upper arm bone), helping you to raise and rotate your arm.
There is a lubricating sac called a bursa between the rotator cuff and the bone on top of the shoulder (acromion). The bursa allows the rotator cuff tendons to glide freely when you move your arm. When the rotator cuff tendons are injured or damaged, this bursa can also become inflamed and painful.
Description

When one or more of the rotator cuff tendons is torn, the tendon becomes partially or completely detached from the head of the humerus.
Illustration of a rotator cuff tendon torn away from bone
In most rotator cuff tears, the tendon is torn away from the bone.
Most tears occur in the supraspinatus tendon, but other parts of the rotator cuff may also be involved.
In many cases, torn tendons begin by fraying. As the damage progresses, the tendon can completely tear, sometimes with lifting a heavy object.
There are different types of tears.
Partial tear. This type of tear does not completely detach the tendon from the bone. It is called partial because the tear goes only partially through the thickness of the tendon. The tendon is still attached to the bone, but it is thinned.
Full-thickness tear. With this type of tear, there is detachment of part of the tendon from the bone.
When only a small part of the tendon is detached from the bone, it is referred to as a full-thickness incomplete tear.
When a tendon is completely detached from the bone, it is referred to as a full-thickness complete tear. With a full-thickness complete tear, there is basically a hole in the tendon.
Rotator cuff tendons and a full-thickness tear in the supraspinatus tendon
(Left) Overhead view of the four tendons that form the rotator cuff.
(Right) A full-thickness tear in the supraspinatus tendon.
Front view of rotator cuff and full-thickness tear in supraspinatus tendon
(Left) The front view of a normal rotator cuff.
(Right) A full-thickness tear in the supraspinatus tendon.
Cause

There are two main causes of rotator cuff tears: injury and wear (degeneration).
Acute Tear

If you fall down on your outstretched arm or lift something too heavy with a jerking motion, you can tear your rotator cuff. This type of tear can occur with other injuries, such as a broken collarbone, a dislocated shoulder, or a wrist fracture.
Degenerative (Wear-Related) Tear

Most tears are the result of a wearing down of the tendon that occurs slowly over time. This degeneration naturally occurs as we age and in most cases is relatively painless.
Rotator cuff tears are more common in the dominant arm — the arm you prefer to use for most tasks. If you have a degenerative tear in one shoulder, there is a greater likelihood of a rotator cuff tear in the opposite shoulder — even if you have no pain in that shoulder.
Several factors contribute to degenerative, or chronic, rotator cuff tears.
Repetitive stress. Repeating the same shoulder motions again and again can stress your rotator cuff muscles and tendons. Baseball, tennis, rowing, and weightlifting are examples of activities that can put you at risk for overuse tears. Many jobs and routine chores can cause overuse tears, as well.
Lack of blood supply. As we get older, the blood supply in our rotator cuff tendons lessens. Without a good blood supply, the body's natural ability to repair tendon damage is impaired. This can ultimately lead to a tendon tear.
Risk Factors

Because most rotator cuff tears are largely caused by the normal wear and tear that goes along with aging, people over 40 are at greater risk.
People who do repetitive lifting or overhead activities are also at risk for rotator cuff tears. Athletes are especially vulnerable to overuse tears, particularly tennis players and baseball pitchers. Painters, carpenters, and others who do overhead work also have a greater chance for tears.
Although overuse tears caused by sports activity or overhead work also occur in younger people, most tears in young adults are caused by a traumatic injury, like a fall.
Related Articles
TREATMENT
Rotator Cuff Tears: Surgical Treatment Options
DISEASES & CONDITIONS
Rotator Cuff Tears: Frequently Asked Questions
TREATMENT
Shoulder Arthroscopy
DISEASES & CONDITIONS
Shoulder Impingement/Rotator Cuff Tendinitis
Symptoms

The most common symptoms of a rotator cuff tear include:
Pain at rest and at night, particularly if lying on the affected shoulder
Pain when lifting and lowering your arm or with specific movements
Weakness when lifting or rotating your arm
Crepitus, or a crackling sensation, when moving your shoulder in certain positions
Tears that happen suddenly, such as from a fall, usually cause intense pain. There may be a snapping sensation and immediate weakness in your upper arm.
Tears that develop slowly due to overuse may also cause pain and arm weakness. You may have pain in the shoulder when you lift your arm, or pain that moves down your arm.
At first, the pain may be mild and present only when lifting your arm over your head, such as reaching into a cupboard. Over-the-counter medication, such as aspirin, ibuprofen, or naproxen, may relieve the pain.
Over time, the pain may become more noticeable at rest and no longer goes away with medications. You may have pain when you lie on the painful side at night. The pain and weakness in the shoulder may make routine activities, such as combing your hair or reaching behind your back, more difficult.
It should be noted that some rotator cuff tears are not painful. These tears, however, may still result in arm weakness and other symptoms.
To Top
Doctor Examination

Medical History and Physical Examination

After discussing your symptoms and medical history, your doctor will examine your shoulder.
They will check to see whether it is tender in any area or whether there is a deformity.
They will have you move your arm in several different directions to measure the range of motion of your shoulder.
They will test your arm strength.
Doctor testing patient's range of motion
Your doctor will test your range of motion by having you move your arm in different directions.
Reproduced with permission from JF Sarwark, ed: Essentials of Musculoskeletal Care, ed 4. Rosemont, IL, American Academy of Orthopaedic Surgeons, 2010.
They will check for other problems with your shoulder joint.
They may also examine your neck to make sure that the pain is not coming from a pinched nerve, and to rule out other conditions, such as arthritis.
Imaging Tests

Other tests which may help your doctor confirm your diagnosis include:
X-rays. The first imaging tests performed are usually X-rays. Because X-rays do not show the soft tissues of your shoulder like the rotator cuff, plain X-rays of a shoulder with rotator cuff pain are usually normal and may show a small bone spur, which is a normal finding. The reason X-rays are done is to make sure you don't have other reasons for your shoulder pain, such as arthritis.
Magnetic resonance imaging (MRI) or ultrasound. An MRI can better show soft tissues, like the rotator cuff tendons, than an X-ray. It can show the rotator cuff tear, as well as where the tear is located within the tendon and the size of the tear. An MRI can also give your doctor a better idea of how old or new a tear is because it can show the quality of the rotator cuff muscles.
Treatment

If you have a rotator cuff tear and keep using it despite increasing pain, you may cause further damage. A rotator cuff tear can get larger over time.
Chronic shoulder and arm pain are good reasons to see your doctor. Early treatment can prevent your symptoms from getting worse. It will also get you back to your normal routine quicker.
The goal of any treatment is to reduce pain and restore function. There are several treatment options for a rotator cuff tear, and the best option is different for every person. In planning your treatment, your doctor will consider:
Your age
Your activity level
Your general health
The type of tear you have
There is no evidence of better results from surgery performed near the time of injury versus later on. For this reason, many doctors first recommend management of rotator cuff tears with physical therapy and other nonsurgical treatments.
Nonsurgical Treatment

In about 80 to 85% of patients, nonsurgical treatment relieves pain and improves function in the shoulder.
Nonsurgical treatment options may include:
Rest. Your doctor may suggest rest and limiting overhead activities.
Activity modification. Avoid any activities that cause shoulder pain.
Nonsteroidal anti-inflammatory drugs (NSAIDs). Anti-inflammatory drugs like ibuprofen, aspirin, and naproxen can reduce pain and swelling.
Strengthening exercises and physical therapy. Specific exercises will restore movement and strengthen your shoulder. Your exercise program will include stretches to improve flexibility and range of motion. Strengthening the muscles that support your shoulder can relieve pain and prevent further injury.
Steroid injection. If rest, medications, and physical therapy do not relieve your pain, an injection of a local anesthetic combined with cortisone may be helpful. Cortisone is a very effective anti-inflammatory medicine; however, it is not effective for all patients. And if it is effective for you, there is no way to know how long the effects will last; it may be weeks, months, years, or possibly the rest of your life. On average, injections can be expected to provide pain relief in about two-thirds of patients for a period of at least 3 months.
Cortisone injection in the shoulder
A cortisone injection may relieve painful symptoms.
The chief advantage of nonsurgical treatment is that it avoids the major risks of surgery, such as:
Infection
Permanent stiffness
Anesthesia complications
Sometimes lengthy recovery time
The disadvantages of nonsurgical treatment are:
The size of the tear may increase over time.
You may need to limit activities.
Surgical Treatment

Your doctor may recommend surgery if your pain does not improve with nonsurgical methods. Continued pain is the main indication for surgery. However, your doctor may also suggest surgery if you are very active and/or use your arms for overhead work or sports.
Other signs that surgery may be a good option for you include:
Your symptoms have lasted 6 to 12 months
You have a large tear (more than 3 cm) and the quality of the surrounding tissue is good
You have significant weakness and loss of function in your shoulder
Your tear was caused by a recent, acute injury
Surgery to repair a torn rotator cuff most often involves re-attaching the tendon to the head of humerus (upper arm bone). There are a few options for repairing rotator cuff tears. Your orthopaedic surgeon will discuss with you the best procedure to meet your individual health needs.

**Original Spanish PEM**

Tenga en cuenta que, debido a la pandemia de la COVID-19, muchos hospitales y sistemas de salud solicitan que los pacientes posterguen sus procedimientos ortopédicos programados. Para obtener más información, consulte Qué hacer si se pospone su cirugía ortopédica.
Un desgarre del manguito rotador es una causa frecuente de dolor y discapacidad en adultos. Todos los años, casi 2 millones de personas en los Estados Unidos consultan al médico debido a un problema con el manguito rotador.
Un manguito rotador desgarrado debilita al hombro. Esto significa que muchas de las actividades diarias, como peinarse el cabello o vestirse, se pueden tornar doloros as y difíciles de hacer.
Anatomía

El hombro está compuesto por tres huesos: el hueso del brazo superior (húmero), el omóplato (escápula) y la clavícula. El hombro es una articulación de bola y cavidad: la bola (o cabeza) del hueso del brazo superior encaja dentro de una cavidad hueca del omóplato.

Esta ilustración del hombro destaca los componentes principales de la articulación.
El brazo se mantiene en la cavidad del hombro gracias al manguito rotador. El manguito rotador es un grupo de cuatro músculos que se unen y junto con los tendones forman una cubierta alrededor de la cabeza del húmero. El manguito rotador une el húmero con el omóplato y ayuda a elevar y rotar el brazo.

Los tendones del manguito rotador cubren la cabeza del húmero (hueso del brazo superior), lo que ayuda a elevar y rotar el brazo.
Hay un saco de líquido lubricante llamado bolsa (o bursa) entre el manguito rotador y el hueso de la parte superior del hombro (acromion). La bolsa (bursa) permite que los tendones del manguito rotador se deslicen con libertad cuando mueve el brazo. Cuando los tendones del manguito rotador se lesionan o dañan, esta bolsa también puede inflamarse y causar dolor.
Descripción

Cuando se desgarra uno o más de los tendones del manguito rotador, el tendón ya no se adhiere completamente a la cabeza del húmero.
La mayoría de los desgarres ocurren en el tendón supraespinoso, pero pueden verse afectadas otras partes del manguito rotador.

En la mayoría de los desgarres del manguito rotador, el tendón se separa del hueso.
En muchos casos, los tendones desgarrados comienzan a deshilacharse. A medida que avanza el daño, el tendón se puede desgarrar por completo, en ocasiones al levantar un objeto pesado.
Existen distintos tipos de desgarres.
Desgarre parcial. Este tipo de desgarre también se llama desgarre incompleto. Daña el tendón, pero no lo corta por completo.
Desgarre total del manguito rotador. Este tipo de desgarre también se llama desgarre completo. Separa todo el tendón del hueso. Con un desgarre total del manguito rotador, básicamente hay un orificio en el tendón.

(Izquierda) Vista superior de los cuatro tendones que conforman el manguito rotador. (Derecha) Desgarre total del manguito rotador en el tendón supraespinoso.

(Izquierda) Vista frontal de un manguito rotador normal. (Derecha) Desgarre total del manguito rotador en el tendón supraespinoso.
Causa

Existen dos causas principales de los desgarres del manguito rotador: lesión y degeneración.
Desgarre grave

Si se cae sobre el brazo estirado o levanta algo demasiado pesado con un movimiento repentino, se puede desagarrar el manguito rotador. Este tipo de desagarre puede ocurrir con otras lesiones del hombro, como la fractura de clavícula o el hombro dislocado.
Desgarre degenerativo

La mayoría de los desgarres son el resultado del desgaste del tendón que se produce lentamente con el transcurso del tiempo. Esta degeneración ocurre de forma natural a medida que envejecemos. Los desgarres del manguito rotador son más frecuentes en el brazo dominante. Si tiene un desgarre degenerativo en uno de los hombros, existe una mayor probabilidad de que tenga un desgarre del manguito rotador en el hombro opuesto, aunque no tenga dolor en ese hombro.
Diversos factores contribuyen a que se produzcan desgarres degenerativos o crónicos del manguito rotador.
Tensión repetitiva. Repetir los mismos movimientos del hombro una y otra vez puede tensionar los músculos y los tendones del manguito rotador. El béisbol, el tenis, el remo y el levantamiento de pesas son ejemplos de actividades deportivas que lo pueden poner en riesgo de sufrir desgarres por uso excesivo. Muchos trabajos y muchas tareas de rutina también pueden causar desgarres por uso excesivo.
Falta de irrigación sanguínea. A medida que envejecemos, disminuye la irrigación sanguínea en el manguito rotador. Sin una irrigación sanguínea adecuada, se deteriora la capacidad natural del cuerpo para reparar el daño del tendón. En última instancia, esto puede producir el desgarre de un tendón.
Espolones óseos. A medida que envejecemos, a menudo se desarrollan espolones óseos (crecimiento excesivo del hueso) en la cara inferior del hueso acromion. Cuando levantamos los brazos, los espolones rozan contra el tendón del manguito rotador. Esta afección se llama pinzamiento del hombro y, con el transcurso del tiempo, debilita el tendón y facilita el desgarre.
Factores de riesgo

Debido a que la mayoría de los desgarres del manguito rotador están causados por el uso y el desgaste normales que ocurren con el envejecimiento, las personas de más de 40 años tienen más riesgo.
Las personas que hacen actividades repetitivas de levantamiento o sobre la altura de la cabeza también tienen riesgo de padecer desgarres del manguito rotador. En especial, los atletas son vulnerables a los desgarres por uso excesivo, particularmente los jugadores de tenis y los lanzadores de béisbol. Los pintores, los carpinteros y las personas que realizan trabajos sobre la altura de la cabeza también tienen más probabilidades de padecer desgarres.
Aunque los desgarres por uso excesivo causados por actividades deportivas o trabajos sobre la altura de la cabeza también se presentan en personas más jóvenes, la mayoría de los desgarres en adultos jóvenes están causados por una lesión traumática, como una caída.
Related Articles
RECOVERY
Rotator Cuff and Shoulder Rehabilitation Exercises
DISEASES & CONDITIONS
Dolor de hombro y problemas comunes del hombro (Shoulder Pain and Common Shoulder Problems)
Síntomas

Los síntomas más comunes del desgarre del manguito rotador incluyen los siguientes:
dolor durante el descanso y por la noche, en particular, si se recuesta sobre el hombro afectado;
dolor cuando levanta o baja el brazo, o con movimientos específicos;
debilidad cuando levanta o rota el brazo;
sensación de crepitación o chasquido cuando mueve el hombro en determinadas posiciones.
Los desgarres que ocurren de manera repentina, como por ejemplo, debido a una caída, por lo general, causan un dolor intenso. Puede haber una sensación de chasquido y debilidad inmediata en el brazo superior.
Los desgarres que se desarrollan lentamente debido al uso excesivo, causan dolor y debilidad del brazo. Puede tener dolor en el hombro cuando levanta el brazo o dolor que lo hace mover el brazo hacia abajo. Al principio, el dolor puede ser moderado y solo presentarse cuando levanta el brazo sobre la altura de la cabeza, como cuando quiere tomar algo de la alacena. Los medicamentos de venta libre, como la aspirina o el ibuprofeno, pueden aliviar el dolor al principio.
Con el transcurso del tiempo, el dolor puede volverse más perceptible durante el descanso y no desaparecer con los medicamentos. Puede tener dolor cuando se recuesta sobre el costado afectado durante la noche. El dolor y la debilidad en el hombro pueden dificultar las actividades cotidianas, como peinarse el cabello o rascarse la espalda.
Cabe mencionar que algunos desgarres del manguito rotador no son dolorosos. Sin embargo, estos desgarres también pueden causar debilidad del brazo y otros síntomas.
To Top
Examen del médico

Historia clínica y examen físico

Después de hablar acerca de los síntomas y la historia clínica, el médico le examinará el hombro. Lo revisará para ver si tiene sensibilidad en la zona o si existe una deformidad. Para medir el rango de movimiento del hombro, el médico le pedirá que mueva el brazo en distintas direcciones. También evaluará la fuerza del brazo.

El médico le pedirá que mueva el brazo en distintas direcciones para evaluar el rango de movimiento.
Reproducido de JF Sarwark, ed.: Essentials of Musculoskeletal Care, ed 4. Rosemont, IL, American Academy of Orthopaedic Surgeons, 2010.
El médico comprobará que no tenga otros problemas en la articulación del hombro. También puede examinarle el cuello para asegurarse de que el dolor no provenga de un “nervio pinzado” y para descartar otras afecciones, como la artritis.
Pruebas por imágenes

Otras pruebas que pueden ayudar al médico a confirmar el diagnóstico incluyen las siguientes:
Radiografías. Por lo general, las primeras pruebas por imágenes que se realizan son las radiografías. Debido a que las radiografías no muestran los tejidos blandos del hombro, como el manguito rotador, las radiografías simples de un hombro con dolor del manguito rotador, por lo general, son normales y pueden mostrar un pequeño espolón óseo.
Imagen por resonancia magnética (IRM) o ecografía. Estos estudios pueden mostrar mejor los tejidos blandos, como los tendones del manguito rotador. Pueden mostrar el desgarre del manguito rotador, así como dónde está ubicado el desgarre, dentro del tendón, y el tamaño del desgarre. Una IRM también puede brindarle al médico una mejor noción de cuán “antiguo” o “nuevo” es un desgarre, debido a que puede mostrar la calidad de los músculos del manguito rotador.
Tratamiento

Si tiene un desgarre del manguito rotador y lo continúa usando a pesar de que el dolor es cada vez más intenso, puede provocar un daño mayor. Un desgarre del manguito rotador se puede agrandar con el transcurso del tiempo.
El dolor crónico en el hombro y el brazo es un buen motivo para consultar al médico. El tratamiento temprano puede prevenir que empeoren los síntomas. También le permitirá volver a la rutina normal mucho más rápido.
El objetivo de todo tratamiento es disminuir el dolor y restaurar la función. Existen diversas opciones de tratamiento para el desgarre del manguito rotador, y la mejor opción varía según cada persona. Al planificar su tratamiento, el médico tendrá en cuenta la edad, el nivel de actividad, la salud general y el tipo de desgarre que tiene.
No existe evidencia de que los resultados sean mejores con una cirugía realizada cerca del momento de la lesión en comparación con una cirugía posterior. Por este motivo, los médicos primero recomiendan tratar los desgarres del manguito rotador con terapia física y otros tratamientos no quirúrgicos.
Tratamiento no quirúrgico

En aproximadamente el 80% de los pacientes, el tratamiento no quirúrgico alivia el dolor y mejora la función del hombro.
Las opciones de tratamiento no quirúrgico pueden incluir las siguientes:
Descanso. El médico puede sugerirle que descanse y limite los movimientos sobre la altura de la cabeza. También le puede recetar un cabestrillo para ayudarlo a proteger el hombro y mantenerlo inmóvil.
Modificación de la actividad. Evite las actividades que causan dolor en el hombro.
Medicamentos antiinflamatorios no esteroideos. Los medicamentos como el ibuprofeno y el naproxeno disminuyen el dolor y la inflamación.
Ejercicios de fortalecimiento y terapia física. Los ejercicios específicos restaurarán el movimiento y fortalecerán el hombro. El programa de ejercicios incluirá estiramientos para mejorar la flexibilidad y el rango de movimiento. El fortalecimiento de los músculos que sostienen el hombro puede aliviar el dolor e impedir una lesión en el futuro.
Inyección de esteroides.Si el descanso, los medicamentos y la terapia física no alivian el dolor, pueden ser útiles una inyección de anestesia local y una preparación de cortisona. La cortisona es un medicamento antiinflamatorio muy eficaz. Sin embargo, no es eficaz para todos los pacientes.

Una inyección de cortisona puede aliviar los síntomas dolorosos.
La principal ventaja del tratamiento no quirúrgico es que evita los riegos mayores de la cirugía, como por ejemplo:
infección;
rigidez permanente;
complicaciones de la anestesia;
el tiempo de recuperación que, a veces, es prolongado.
Las desventajas del tratamiento no quirúrgico son las siguientes:
el tamaño del desgarre puede aumentar con el transcurso del tiempo;
las actividades se pueden ver limitadas.
Tratamiento quirúrgico

El médico puede recomendarle cirugía si el dolor no se alivia con métodos no quirúrgicos. El dolor continuo es la principal indicación para una cirugía. Si es muy activo y usa los brazos sobre la altura de la cabeza o hace deportes, el médico también puede recomendarle la cirugía.
Otros signos de que la cirugía puede ser una buena opción para su caso son los siguientes:
los síntomas han durado de 6 a 12 meses;
tiene un desgarre grande (más de 3 cm) y la calidad del tejido circundante es buena;
tiene debilidad y pérdida de la función significativas en el hombro;
una lesión reciente y grave causó el desgarre.
En la mayoría de los casos, la cirugía para reparar un maguito rotador implica volver a unir el tendón a la cabeza del húmero (hueso del brazo superior). Existen algunas pocas opciones para reparar los desgarres del manguito rotador. El cirujano ortopédico hablará con usted acerca del mejor procedimiento para satisfacer sus necesidades de salud particulares.

**AI-Translated PEM (from English to Spanish)**

Desgarro del Manguito Rotador

Un desgarro del manguito rotador es una causa común de dolor y discapacidad en el hombro en los adultos. Cada año, casi 2 millones de personas en los Estados Unidos visitan a sus médicos debido a desgarros del manguito rotador.

Un manguito rotador desgarrado puede debilitar el hombro. Esto significa que muchas actividades diarias, como peinarse o vestirse, pueden volverse dolorosas y difíciles de realizar.

Anatomía

El hombro es una articulación esférica compuesta por tres huesos:

Húmero (hueso de la parte superior del brazo)

Escápula (omóplato)

Clavícula (hueso de la clavícula)

La cabeza del húmero encaja en una cavidad poco profunda de la escápula.

Anatomía normal del hombro

Esta ilustración del hombro destaca los principales componentes de la articulación.

El manguito rotador mantiene el brazo en la cavidad del hombro. Es un grupo de cuatro músculos que se unen como tendones para formar una cubierta alrededor de la cabeza del húmero. El manguito rotador conecta el húmero con la escápula y ayuda a levantar y rotar el brazo.

Tendones del manguito rotador

Los tendones del manguito rotador cubren la cabeza del húmero, ayudando a levantar y rotar el brazo.

Entre el manguito rotador y el hueso superior del hombro (acromion), hay una bolsa lubricante llamada bursa. La bursa permite que los tendones del manguito rotador se deslicen libremente cuando mueves el brazo. Cuando los tendones del manguito rotador se lesionan o dañan, esta bursa también puede inflamarse y volverse dolorosa.

Descripción

Cuando uno o más de los tendones del manguito rotador se desgarran, el tendón se separa parcial o completamente de la cabeza del húmero.

En la mayoría de los desgarros del manguito rotador, el tendón se separa del hueso.

La mayoría de los desgarros ocurren en el tendón del supraespinoso, aunque otras partes del manguito rotador también pueden estar involucradas.

En muchos casos, los tendones desgarrados comienzan a deshilacharse. A medida que el daño avanza, el tendón puede romperse por completo, a veces al levantar un objeto pesado.

Existen diferentes tipos de desgarros:

Desgarro parcial: el tendón no se separa completamente del hueso. El desgarro solo atraviesa parcialmente el grosor del tendón.

Desgarro de espesor completo: parte del tendón se desprende del hueso.

Si solo una pequeña parte se desprende, se denomina desgarro de espesor completo incompleto.

Si el tendón se desprende completamente del hueso, se denomina desgarro de espesor completo total. En este caso, hay un agujero en el tendón.

Causas

Existen dos causas principales de los desgarros del manguito rotador: lesión y desgaste (degeneración).

Desgarro agudo

Si caes sobre el brazo extendido o levantas algo demasiado pesado con un movimiento brusco, puedes desgarrar el manguito rotador. Este tipo de desgarro puede ocurrir junto con otras lesiones, como fractura de clavícula, dislocación del hombro o fractura de muñeca.

Desgarro degenerativo (por desgaste)

La mayoría de los desgarros ocurren debido a un desgaste gradual del tendón con el tiempo. Este proceso es parte del envejecimiento y, en muchos casos, es relativamente indoloro.

Los desgarros del manguito rotador son más comunes en el brazo dominante. Si tienes un desgarro degenerativo en un hombro, existe una mayor probabilidad de que también ocurra en el otro hombro, incluso si no tienes dolor en ese lado.

Varios factores contribuyen a los desgarros crónicos del manguito rotador:

Estrés repetitivo: ciertos deportes y trabajos pueden generar sobrecarga en los tendones, como el béisbol, tenis, remo, levantamiento de pesas, pintura y carpintería.

Falta de irrigación sanguínea: con la edad, la irrigación sanguínea a los tendones del manguito rotador disminuye, lo que reduce la capacidad del cuerpo para reparar el daño.

Factores de riesgo

Dado que la mayoría de los desgarros del manguito rotador son causados por el envejecimiento, las personas mayores de 40 años tienen un mayor riesgo.

Aquellos que realizan levantamientos repetitivos o actividades por encima de la cabeza también están en riesgo. Los atletas, especialmente los jugadores de tenis y lanzadores de béisbol, son más propensos a desgarros por sobreuso. Los pintores, carpinteros y otros trabajadores manuales también tienen un mayor riesgo.

En los adultos jóvenes, la mayoría de los desgarros son causados por una lesión traumática, como una caída.

Síntomas

Los síntomas más comunes de un desgarro del manguito rotador incluyen:

Dolor en reposo y por la noche, especialmente al acostarse sobre el hombro afectado.

Dolor al levantar o bajar el brazo.

Debilidad al levantar o rotar el brazo.

Sensación de crujido (crepitación) al mover el hombro en ciertas posiciones.

Los desgarros repentinos, como los causados por una caída, generalmente provocan un dolor intenso, una sensación de chasquido y debilidad inmediata en el brazo.

Los desgarros por desgaste pueden causar dolor progresivo. Al principio, el dolor puede ser leve y solo aparecer al levantar el brazo sobre la cabeza. Con el tiempo, el dolor puede volverse constante y dificultar actividades diarias como peinarse o vestirse.

Es importante destacar que algunos desgarros del manguito rotador no causan dolor, pero pueden generar debilidad y otras limitaciones.

Examen médico

Tu médico evaluará tu hombro con un historial clínico y examen físico. Se examinará la sensibilidad, rango de movimiento, fuerza y posibles deformidades. También podría evaluar el cuello para descartar problemas nerviosos o artritis.

Pruebas de imagen

Radiografías: no muestran el manguito rotador, pero pueden detectar otras causas del dolor, como artritis.

Resonancia magnética (RM) o ultrasonido: pueden mostrar el estado de los tendones y la ubicación exacta del desgarro.

Tratamiento

Si tienes un desgarro del manguito rotador y continúas usando el brazo a pesar del dolor, puedes agravar la lesión con el tiempo.

El objetivo del tratamiento es reducir el dolor y restaurar la función. Las opciones incluyen:

Tratamiento no quirúrgico (80-85% de éxito)

Reposo y limitación de actividades.

Modificación de actividad para evitar movimientos dolorosos.

Medicamentos antiinflamatorios (AINEs): ibuprofeno, aspirina o naproxeno.

Fisioterapia: ejercicios de estiramiento y fortalecimiento del hombro.

Inyección de cortisona: alivia el dolor en algunos pacientes.

Las ventajas del tratamiento no quirúrgico incluyen evitar los riesgos de la cirugía, como infecciones, rigidez permanente o complicaciones con la anestesia. Sin embargo, el desgarro podría aumentar de tamaño con el tiempo.

Tratamiento quirúrgico

La cirugía puede ser recomendada si:

El dolor persiste tras 6 a 12 meses de tratamiento no quirúrgico.

El desgarro es grande (>3 cm) y el tejido circundante está en buen estado.

Hay debilidad significativa en el hombro.

El desgarro fue causado por una lesión reciente.

El procedimiento más común consiste en volver a unir el tendón a la cabeza del húmero. Tu cirujano ortopédico discutirá la mejor opción para tu caso.
